# An Unfortunate Union: A Case of Multiple Magnet Ingestion in a Pediatric Patient

**DOI:** 10.7759/cureus.21490

**Published:** 2022-01-22

**Authors:** Shane G Stephenson, Colton T Knight, Hunaid N Rana, Todd Standley, Suzy Figarola

**Affiliations:** 1 Radiology, University of South Alabama, Mobile, USA; 2 Pediatric Radiology, University of South Alabama, Mobile, USA

**Keywords:** abdominal radiograph, foreign body radiology, gi radiology, foreign body algorithm, multiple magnet ingestion, pediatric surgery, pediatric foreign body, pediatrics, magnets ingestion, swallowed foreign body

## Abstract

Magnets are among the most dangerous foreign objects that a child can ingest. If more than one magnet is ingested, the attraction between loops of the bowel can bring adjacent loops closer together, leading to perforation, obstruction, or fistulization. Pediatric magnet ingestion patients often require endoscopic or surgical intervention to retrieve the objects and repair the damage created by the magnets. Due to the risks of surgical intervention, management is done with strict adherence to the rare earth magnet ingestion algorithm. We highlighted a pediatric case of multiple magnet ingestion, and the steps that were taken to manage the patient. Our case highlights the potential for complications and the importance of adherence to the management algorithm in these patients. Epidemiology, mechanisms, algorithms, and outcomes for pediatric magnet ingestion patients were discussed.

## Introduction

Ingestion of foreign bodies is common in children, and most foreign bodies will pass spontaneously without the need for intervention [1]. More than 100,000 cases of pediatric foreign body ingestions occur each year with approximately 98% being accidental. The most common objects ingested in the pediatric population include coins, toys, sharp objects, batteries, bones, and food [2]. However, a subset of foreign bodies can become trapped and cause serious injuries. Specifically, ingestion of magnets can cause injury via proximate attraction through the intestinal wall, causing adjacent loops to come in close proximity [3]. Magnet ingestion incurs an extremely high risk of bowel obstruction, perforation, or fistulization following attraction between multiple loops of the bowel. Neodymium or rare-earth magnets, composed of iron, boron, and neodymium, are five to ten times more powerful than traditional magnets and increase the risk of injury. These magnets have been used in increasingly more children’s toys and household products in recent years [4]. Pediatric patients presenting with magnet ingestion often require endoscopic or surgical intervention. In the case of multiple magnet ingestion, laparoscopy or even open abdominal surgical intervention may be required to remove the objects [5]. Hereby we present a complicated case of multiple magnet ingestion in a young child and discuss the proper management of such cases.

## Case presentation

A two-year-old female presented to the emergency department (ED) with her mother at 9:40 P.M. two hours following ingestion of six, spherical, <1 cm magnets. The mother witnessed the patient ingest at least two magnets before she could take them away. The patient last ate one hour after ingestion of the magnets. She was taken to the ED one hour later, where the mother denied abdominal pain, emesis, or choking. She also denied any previous similar incidents. The patient had no significant past medical history, and the physical exam findings were unremarkable.

**Figure 1:**
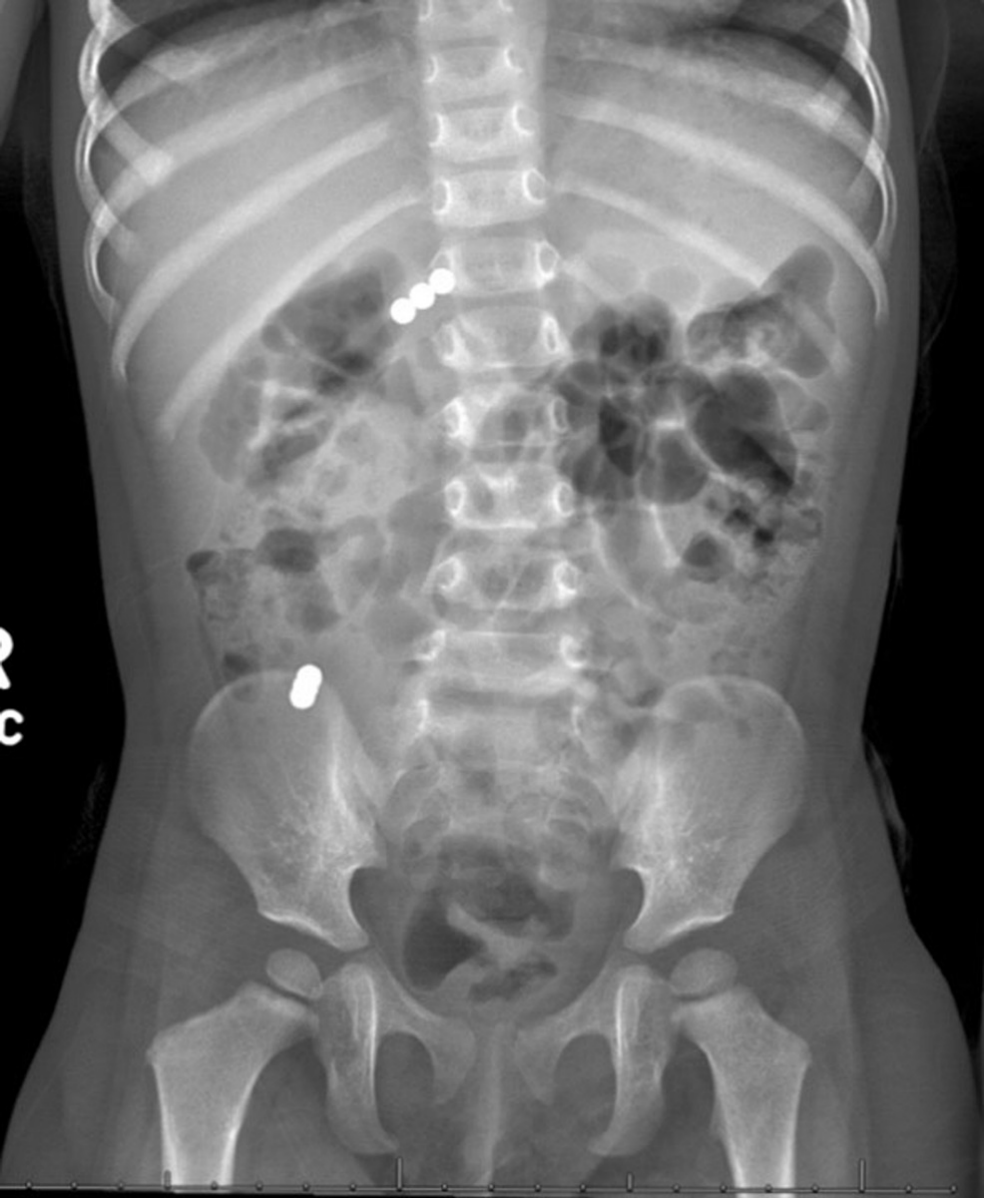
Initial anterior radiograph showing two clusters of three 5 mm spherical magnets in the right abdomen.

At the ED, an X-ray (XR) foreign body examination of the nose to the rectum was obtained. The report mentioned, “There are three clustered spherical radiodense foreign bodies measuring 5 mm in diameter each, located in the right upper abdomen. A similar cluster of three foreign bodies is seen in the right lower abdomen” (Figure 1). Pediatric gastroenterology was consulted and mentioned possible endoscopy in the morning if the magnets were still in an accessible position. Pediatric surgery was also consulted and had no further recommendations as the patient was currently asymptomatic with no acute concerns. The patient was admitted to the pediatric inpatient service for observation following the pediatric foreign body ingestion algorithm that night. Per the algorithm, the patient was followed clinically with serial X-rays repeated every eight to twelve hours, and she was placed on nothing by mouth (NPO) status in case surgical intervention was warranted.

**Figure 2:**
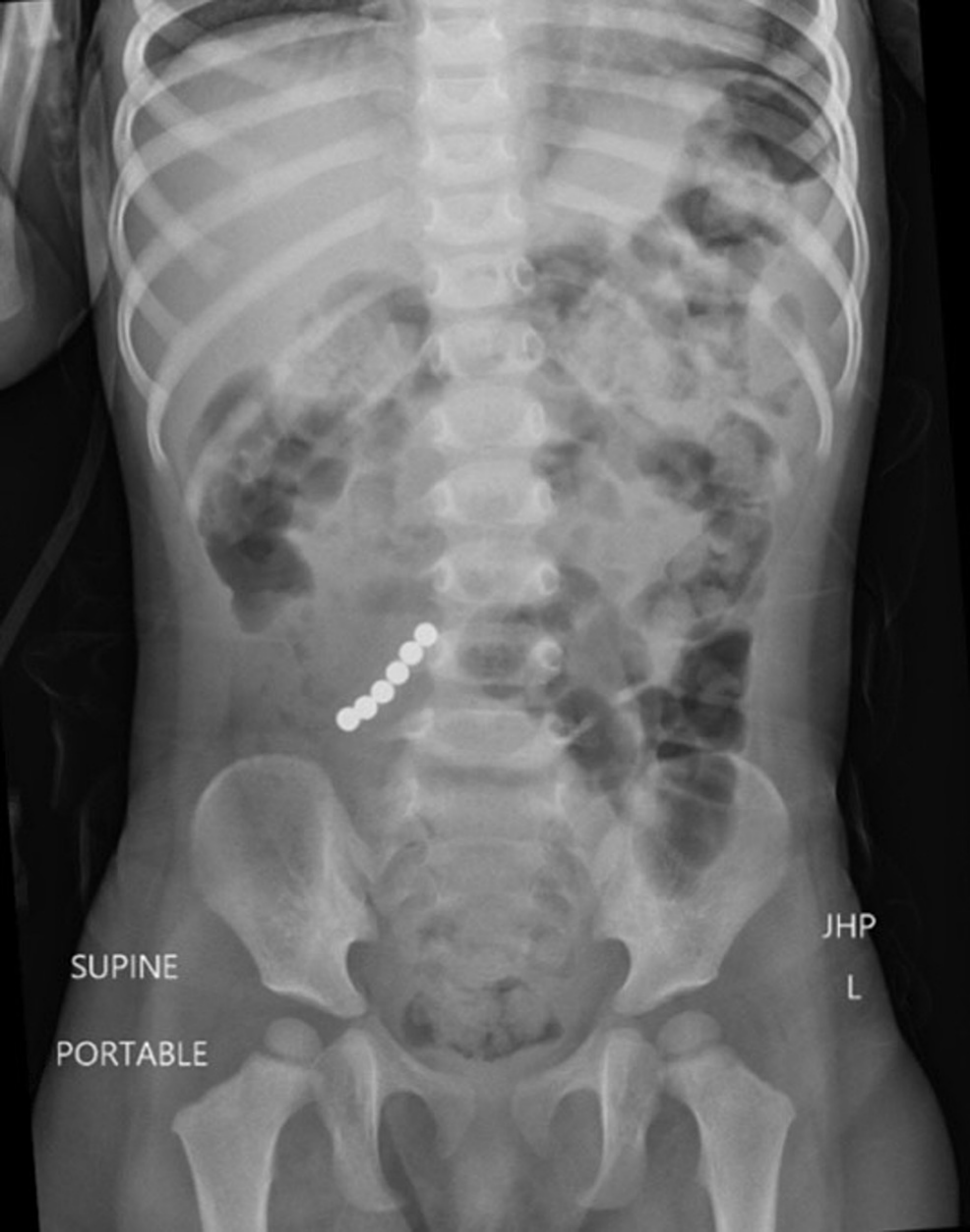
Repeat anterior radiograph showing alignment of all six magnets just right of midline, but no obstructive bowel gas pattern.

**Figure 3:**
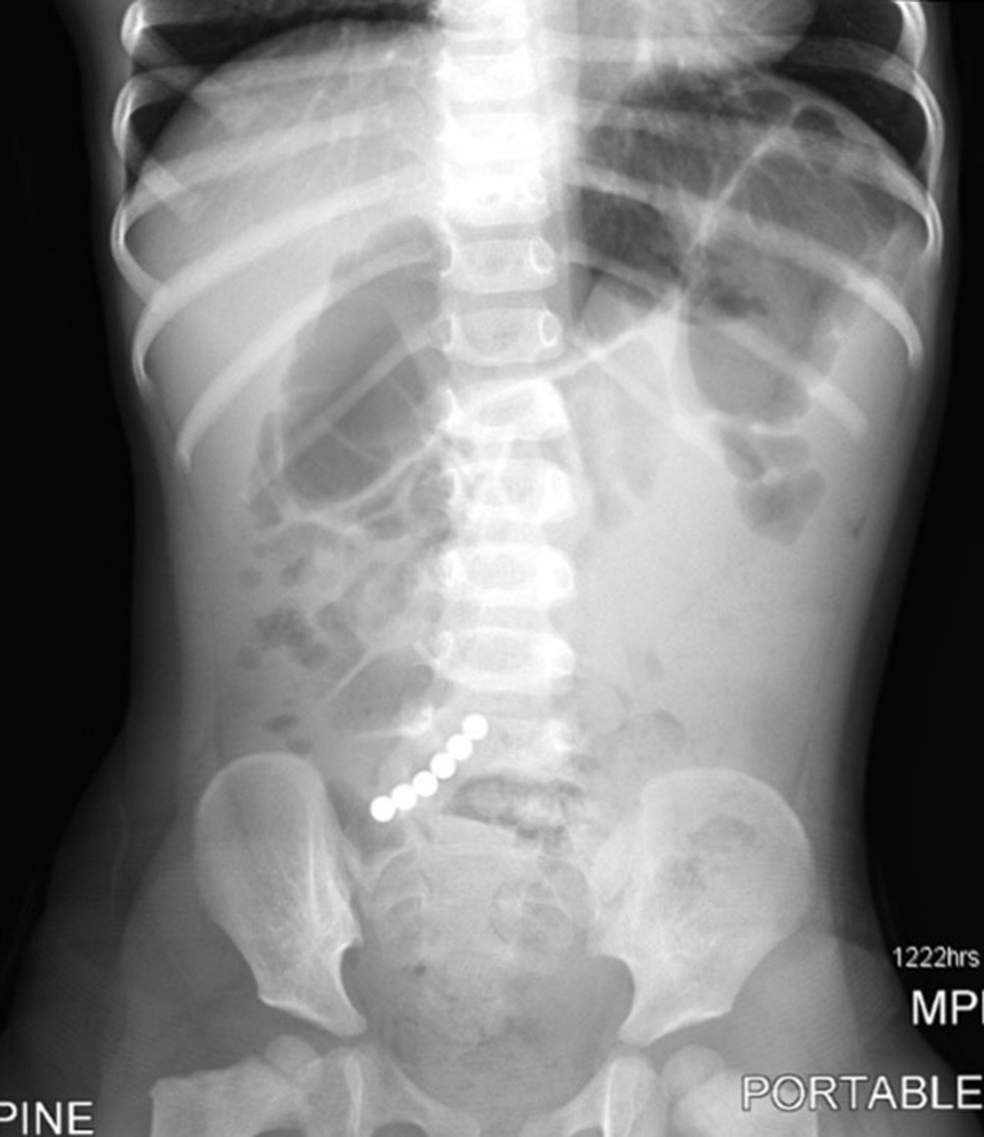
Repeat anterior radiograph showing alignment of all six magnets with an obstructive bowel gas pattern.

**Figure 4:**
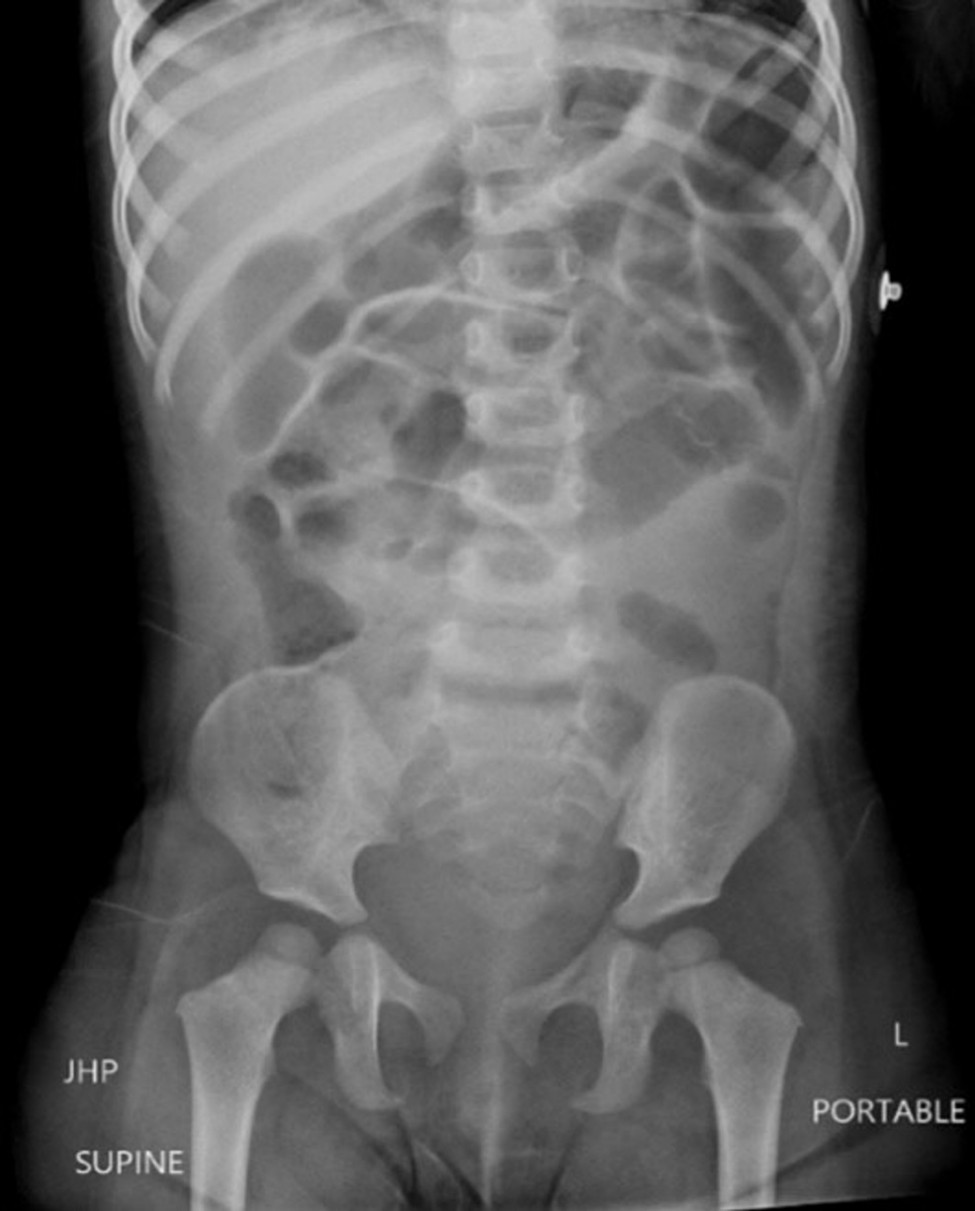
Repeat anterior radiograph following surgical removal of magnets. Magnets can no longer be visualized. There is residual post-operative pneumoperitoneum and gaseous distention of the bowel.

The next morning, another abdominal X-ray was performed. It revealed alignment of the six magnets projecting just right of midline over the right abdomen with a non-obstructive bowel gas pattern (Figure 2). That afternoon, a repeat X-ray was performed, demonstrating the same pattern of alignment. However, mild dilated loops of the bowel were observed within the right and mid-abdomen, indicating a possible bowel obstruction (Figure 3). Diagnostic laparotomy was scheduled for that evening in order to remove the magnets and repair any bowel injury. During the surgery, two enterotomies were performed indicating impending fistulization of bowel between magnets in adjacent segments. Two of the six magnets were removed from the first enterotomy encountered within the cecum and suspected to have traveled through a fistulous tract. Fluoroscopic guidance was then used to identify the location of the remaining four magnets. These magnets were removed following identification of a full-thickness injury in that segment of the small bowel. A partial small bowel resection, repair of cecal injury, and appendectomy were performed. The patient tolerated the procedure well with no immediate complications. X-ray control following surgery confirmed the absence of any foreign bodies (Figure 4), and the patient was discharged on post-operative day three. 

 

Seven days post-surgery, the patient presented back to the ED with chills, redness, and swelling around her surgical incision. She arrived afebrile but tachycardic, and surgical site infection was suspected. She was taken to the operating room (OR) emergently for incision and drainage of a periumbilical abscess. In the OR, the infraumbilical incision was re-opened and purulent fluid was drained. Additionally, multiple perforations were observed in the ileum, jejunum, and cecum. The perforations were repaired, a wedge resection of the terminal ileum was performed, and a drain was placed. The patient was placed on IV antibiotics and continued to be observed. Four days later, the drain was removed but purulence was again noticed around the periumbilical incision. The wound was packed with iodoform gauze, and observation continued. Three days later, the patient again was taken to the OR for washout of the incision and placement of a wound vac. Finally, one week after that, the patient had the wound vac removed and the incision was closed. The next day, the patient was deemed fit for discharge due to the fact that she was tolerating solid food and having appropriate bowel and bladder function.

## Discussion

Magnets are among the most dangerous and potentially life-threatening foreign bodies that a child can ingest. Between the years of 2010 and 2015, an estimated 14,586 children reported to the ED in the United States with suspected magnet ingestion, a significant increase from preceding years [6]. Magnet ingestion can lead to pressure necrosis causing intestinal wall trauma, obstruction, and perforation leading to life-threatening circumstances [7-8]. The risks associated with magnet ingestion have been accentuated by the emergence of commercially available magnets manufactured with boron, iron, and neodymium that produce forces five to ten times stronger than traditional iron magnets [4, 9]. Because many of these new magnets are quite small, ingestion of a single magnet usually passes without complication. However, upon approximation of multiple magnets, pressure necrosis between multiple loops of the bowel can occur. Due to this large force, enteroenteric fistulae may also occur [8-9]. In some extreme circumstances, perforation can progress to peritonitis and further complicate the case. In cases with severe complications such as perforation or peritonitis, surgical intervention is mandatory [7].

**Figure 5:**
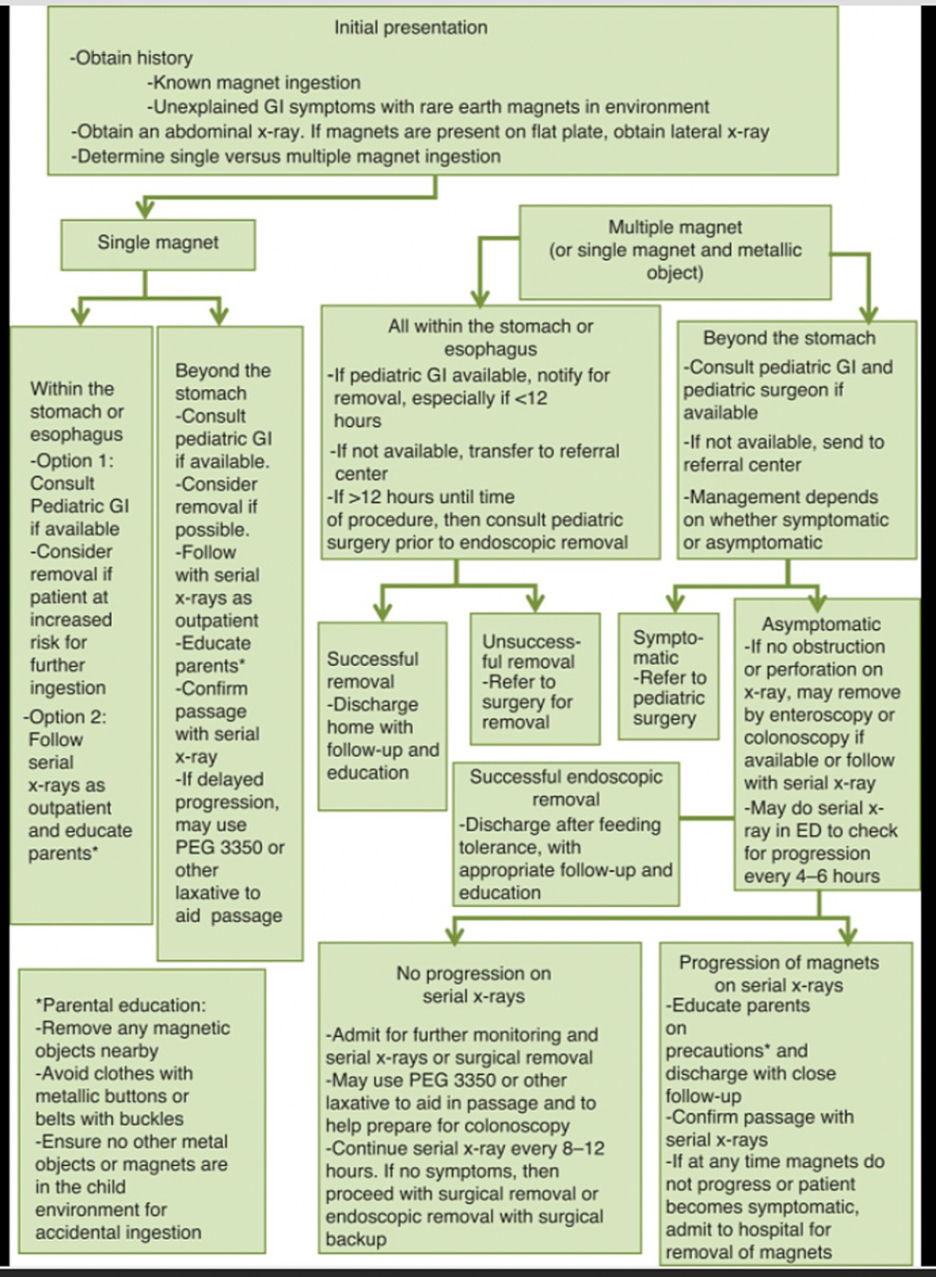
NASPGHAN rare-earth magnet ingestion algorithm. [4]

In 2012, a survey of the North American Society of Pediatric Gastroenterology, Hepatology, and Nutrition (NASPGHAN) members indicated that magnet ingestion was producing more morbidity than initially expected. For this reason, a new algorithm for the management of these patients was created to maximize the positive outcomes [4] (Figure 5). Management can also be complicated by the fact that many patients will be asymptomatic from the time of ingestion until a complication occurs [10]. Once complication occurs, it may be too late for non-invasive management. Therefore, adherence to the management algorithm is imperative for the proper management of pediatric magnet ingestion. The first step of the algorithm upon patient presentation is to obtain a history either of known magnet ingestion or unexplained GI symptoms. Next, an anterior posterior (AP) and lateral abdominal X-ray is obtained to try to visualize the location and number of foreign bodies ingested. It is imperative to obtain multiple X-ray views in order to visualize multiple magnets given that more than one magnet stuck together may be mistaken for a single magnet from only one view. If a single magnet was ingested, conservative management with patient education is warranted. In the case of multiple magnet ingestion, a more comprehensive approach is needed. If all the magnets are located in the stomach or esophagus, endoscopic retrieval is the first choice with subsequent surgery consultation in an unsuccessful attempt. However, if the magnets are located beyond the stomach, the patient’s symptomology becomes important. If there are significant gastrointestinal (GI) symptoms, the patient will immediately be referred to pediatric surgery for removal. However, if asymptomatic, the patient can be followed with serial X-rays every eight to twelve hours looking for progression, perforation, or obstruction. If progression of the magnets occurs, the patient may be discharged with close follow-up, education, and confirmation of passage with serial X-rays. If there is no progression, the patient will be admitted to the hospital for close monitoring and serial X-rays until surgery is warranted [4]. In our patient’s case, the mother was able to visualize the ingestion of the magnets, but the patient was asymptomatic. Multiple magnet ingestion beyond the stomach was confirmed via abdominal X-ray, and the progression of the magnets was observed with serial radiographs. Once progression of the magnets ceased and obstruction was suspected, the patient was taken to surgery. Our case illustrates the potential complications of the surgery, and therefore why it should be the last resort, as indicated in the algorithm.

Because of the tendency to cause bowel injury and severe consequences, the outcomes of pediatric magnet ingestion are typically worse than many other foreign bodies. According to data gathered by the United States Consumer Product Safety Commission, between 2000 and 2012, surgery was required in 69.7% of pediatric magnet ingestion cases and 73.6% of patients were admitted to the hospital. Additionally, 34.3% of patients had multiple bowel perforations or necrosis and 3% experienced fistulization of the bowel [11]. These outcomes highlight the importance of family education and rapid medical guidance in the case of pediatric magnet ingestion.

## Conclusions

We highlighted a case of pediatric magnet ingestion, including the presentation, management, and outcomes. Epidemiology, mechanisms, management, and outcomes were discussed for pediatric magnet ingestion cases. The importance of adherence to the management algorithm and patient education was demonstrated by the epidemiology and considerable consequences seen in our case and many others.
